# NAB-paclitaxel and gemcitabine in metastatic pancreatic ductal adenocarcinoma (PDAC): from clinical trials to clinical practice

**DOI:** 10.1186/s12885-016-2671-9

**Published:** 2016-09-02

**Authors:** Ferdinando De Vita, Jole Ventriglia, Antonio Febbraro, Maria Maddalena Laterza, Alessio Fabozzi, Beatrice Savastano, Angelica Petrillo, Anna Diana, Guido Giordano, Teresa Troiani, Giovanni Conzo, Gennaro Galizia, Fortunato Ciardiello, Michele Orditura

**Affiliations:** 1 Division of Medical Oncology, Department of Internal and Experimental Medicine “F. Magrassi”, Second University of Naples - School of Medicine, c/o II Policlinico, Via Pansini, 5, 80131 Naples, Italy; 2 Division of Medical Oncology, Fatebenefratelli Hospital, Viale Principe di Napoli 14/a, 82100 Benevento, Italy; 3 Divisions of Surgical Oncology, Department of Anesthesiological, Surgical and Emergency Sciences, Second University of Naples - School of Medicine, c/o II Policlinico, Via Pansini, 5, 80131 Naples, Italy

**Keywords:** Metastatic pancreatic cancer, Combination chemotherapy, Nab-paclitaxel, Gemcitabine

## Abstract

**Background:**

Pancreatic adenocarcinoma is an aggressive disease with poor prognosis. In a randomized phase III trial, combination of Nab-paclitaxel (Nab-P) plus gemcitabine showed superior activity and efficacy in first-line treatment compared with gemcitabine alone.

**Methods:**

Nab-P is not dispensed in Italy; however, we obtained this drug from our Ethics Committee for compassionate use. The aim of this study was to evaluate the efficacy and safety profile of this Nab-P and gemcitabine combination in a cohort of patients treated outside clinical trials. From January 2012 to May 2014, we included 41 patients with advanced pancreatic adenocarcinoma receiving combination of 125 mg/m^2^ Nab-P and 1 g/m^2^ gemcitabine on days 1, 8 and 15 of a 28-day cycle, as first-line treatment. Median age of patients was 67 (range 41–77) years, and 11 patients were aged ≥70 years.

**Results:**

Eastern Co-operative Oncology Group performance status was 0 or 1 in 32 patients (78 %) and 2 in nine patients (22 %). Primary tumor was located in the pancreatic head or body/tail in 24 (58.5 %) and 17 (41.5 %) patients, respectively, and nine patients had received biliary stent implantation before starting chemotherapy. Median carbohydrate antigen 19–9 level was 469 U/l (range 17.4–61546 U/l) and 29 patients (70.7 %) had referred pain at the time of diagnosis. Patients received a median six cycles (range 1–14) of treatment. Overall response rate was 36.6 %; median progression-free survival was 6.7 months [(95 % confidence interval (CI) 5.966–8.034), and median overall survival was 10 months (95 % CI 7.864–12.136). Treatment was well tolerated. No grade 4 toxicity was reported. Grade 3 toxicity included neutropenia in 10 patients (24.3 %), thrombocytopenia in five (12 %), anemia in three (7.3 %), diarrhea in four (9.7 %), nausea and vomiting in two (4.9 %), and fatigue in six (14.6 %). Finally, pain control was achieved in 24 of 29 patients (82.3 %) with a performance status improvement of 10 % according to the Karnofsky scale.

**Conclusions:**

Our results confirm that combination of gemcitabine plus Nab-P is effective both in terms of overall response rate, progression-free survival and overall survival, with a good safety profile.

## Background

Pancreatic adenocarcinoma represents ~3 % of newly diagnosed cancers annually worldwide [1]. It is an aggressive disease and despite the efforts of the past few decades, the 5-year overall survival (OS) rate remains poor and does not exceed 5 % [2]. Most patients are diagnosed with locally advanced or metastatic disease, and their only treatment approach is palliative chemotherapy [3]. Since 1997, single-agent gemcitabine has been regarded as first-line standard of care in metastatic disease. However, subsequently, most gemcitabine-based chemotherapy regimens have not been able to improve OS significantly when compared with alone [4–10]. Only recently, the four-drug regimen FOLFIRINOX (oxaliplatin, irinotecan, fluorouracil and leucovorin) has been shown to improve the objective response rate (ORR), progression-free survival (PFS) and OS compared with single agent gemcitabine, albeit with a significant toxicity profile [11]. In preclinical studies, albumin-bound paclitaxel particles (Nab-P) have been shown to exert anti-tumor activity as a single agent and synergistic activity in combination with gemcitabine in murine models of pancreatic cancer [12, 13]. On the basis of preclinical evidence, a phase I–II clinical trial of Nab-P combined with gemcitabine showed promising results in previously untreated patients with metastatic pancreatic adenocarcinoma, with a median survival of 12.2 months and manageable safety profile [14]. In a subsequent randomized phase III trial involving 861 patients, this combination was demonstrated to improve OS, PFS and ORR significantly over gemcitabine alone, thus establishing the combination of Nab-P and gemcitabine as standard first-line treatment for metastatic pancreatic cancer [15]. However, based on enrollment criteria, the population of the above trial might have not fully mirrored real-life clinical practice. Therefore, we carried out a retrospective analysis to evaluate efficacy and safety profile of this drug combination in a cohort of 41 patients treated outside clinical trials.

## Methods

### Patient population

From January 2012 to May 2014, patients with metastatic pancreatic ductal adenocarcinoma (PDAC), receiving first-line treatment with combination of Nab-P and gemcitabine, were considered eligible for our retrospective analysis. Written informed consent was obtained from each patient before starting treatment in accordance with the Declaration of Helsinky. The ethics committee of Second University of Naples approved the use of Nab-P plus gemcitabine for compassionate use, on the evidence of good results obtaining in phase I/II trial [14] and the use of date for our retrospective analysis. Inclusion criteria were clinicopathologically confirmed PDAC, age ≥18 years, Karnofsky performance status (KPS) ≥60 %, adequate hematological function (neutrophil count ≥1500/mm^3^, platelet count ≥100,000/mm^3^, and hemoglobin ≥ 9 g/dl), adequate hepatic function [total bilirubin <1.5 times the upper limit of normal range (ULN), aspartate aminotransferase (AST) and alanine amino transferase (ALT) ≤2.5 × ULN or AST and ALT ≤5.0 × ULN in case of liver metastases], and adequate renal function. Patients with prior adjuvant gemcitabine treatment were included if treatment was completed at least 6 months before. Data were censored on May 2014. All patients treated with at least one cycle of Nab-P + gemcitabine were included for analysis. The characteristics of the series are summarized in Table 1.

**Table 1:** Baseline patient characteristics

Characteristic	Nab-Paclitaxel plus Gemcitabine (range)
AGE (range)	67 (41–77)
> 70	11 (26,8 %)
SEX	
M	18 (43,9 %)
F	23 (56,1 %)
PS (Karnofsky)	
100 %	18 (43,9 %)
80–90 %	14 (34.1 %)
60–70 %	9 (21,9 %)
PANCREATIC PRIMARY LOCATION	
HEAD	24 (58.5 %)
BODY/TAIL	17 (41.5 %)
SITE OF METASTASIS	
Lung	10 (24,3 %)
Node	7 (17 %)
Liver	30 (73,1 %)
Peritoneum	5 (12,2 %)
Bone	1 (2,4 %)
No OF METASTATIC SITES	
1	30 (73,1 %)
≥ 2	11 (26,9 %)
BILIARY STENT	9 (21.9 %)
MEDIAN CA19.9	469 U/I (17.4–61546)
PREVIOUS SURGERY	11 (26.8 %)
PREVIOUS ADJUVANT GEMCITABINE	10 (24.3 %)

### Treatment schedule

Nab-P, 125 mg/m^2^, followed by 1 g/m^2^ gemcitabine, was administered intravenously on days 1, 8 and 15 every 4 weeks until disease progression or evidence of unacceptable toxicity. Antiemetic prophylaxis with 5-HT3 antagonists plus dexamethasone was used in all patients. Recombinant human granulocyte colony-stimulating factor and erythropoietin were administered as needed. Dose reductions were applied in cases of grade 3/4 toxicity.

### Assessments

Tumor response evaluation was performed every 12 weeks by means of spiral computed tomography. Response Evaluation Criteria In Solid Tumors (RECIST) 1.1 were used [16]. Blood tests were performed at baseline and then at each cycle, while measurement of the carbohydrate antigen (CA)19-9 serum level was performed at baseline and every 12 weeks. Activity was evaluated in terms of ORR [defined as complete response (CR) + partial response (PR)], and disease control rate (defined as CR + PR + stable disease). Six-month clinical benefit rate (CBR) was defined as improvement of at least one of three parameters among KPS, weight loss, and pain, without worsening in any others, in association with sustained DCR (disease control rate) for at least 6 months. Efficacy was evaluated in terms of OS and PFS. The former was defined as the interval between the start of Nab-P and gemcitabine first-line therapy to death or last follow-up visit. The latter was defined as the interval between the start of Nab-P and gemcitabine therapy to clinical progression or death or last follow-up visit if disease did not progress. Safety was monitored by investigators and reported in clinical charts according to Common Toxicity Criteria for Adverse Events (CTCAE) version 4.0. Variables assessed for prognostic correlations included age ≥70 years, sex, KPS, primary tumor site, baseline CA19-9 level ≥59 × ULN, presence of liver metastases, multiple metastatic involvement, 12-week decrease of CA19-9 level ≥50 % from baseline, basal bilirubin level, neutrophil to lymphocyte ratio (NLR), calculated as the absolute neutrophil count divided by the absolute lymphocyte count measured in × 10^3^/ml, and biliary stent implantation.

### Statistical analysis

Survival distribution was estimated by the Kaplan–Meier method with 95 % confidence interval (CI) [17]. Differences in survival according to clinical parameters or treatment were evaluated by the log-rank test and described by the Kaplan–Meier method. For the final analysis, the survival status of all patients was updated within 2 months before the data cutoff of May 2014. Cox proportional-hazards model was applied to multivariate survival analysis. All the significant variables in the univariate model were used to build the multivariate model of survival. SPSS version 20.0 (Chicago, IL, USA) was used for statistical analysis. Values of *p* ≤ 0.05 indicated statistical significance.

## Results

From January 2012 to May 2014, we retrospectively reviewed the clinical records of 41 patients with PDAC.

### Activity

The patients received a median of six cycles (range 1–14) of treatment. Two CRs and 13 PRs were observed for an ORR of 36.6 %. Stable disease was recorded in 14 patients, yielding a global DCR of 70.7 %. Twenty-seven patients reported cancer-related pain or disease-related symptoms before starting treatment; 24 (58.5 %) of them benefitted by at least one point of pain relief according to Numeric Rating Scale or symptom improvement, resulting in a 6-month CBR of 51.2 %. A 12-week decrease of CA19-9 levels ≥50 % from baseline was recorded in 17 patients (41.5 %) (Table 2). Pearson correlation analysis confirmed a positive, linear, statistically significant correlation between CA19-9 decrease ≥50 % from baseline and tumor response (Pearson correlation 0.581, Sig. two-tailed 0.0001).

**Table 2:** Overall response rate

RESPONSE RATE	
RC	2 (4.8 %)
RP	13 (31.7 %)
ORR	15 (36.6 %)
SD	14 (34.2 %)
DCR	26 (70.7)
PD	12 (29.3)
CA19.9 > 50 % reduction	17 (41.5 %)

### Efficacy

**Fig. 1:**
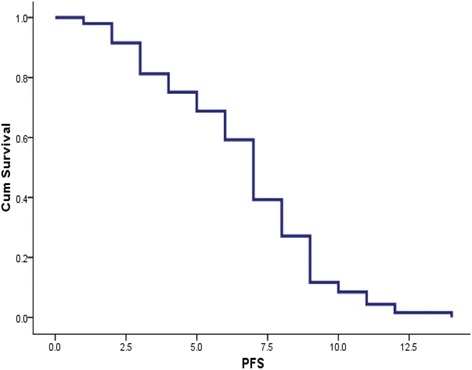
Progression free survival

**Fig. 2:**
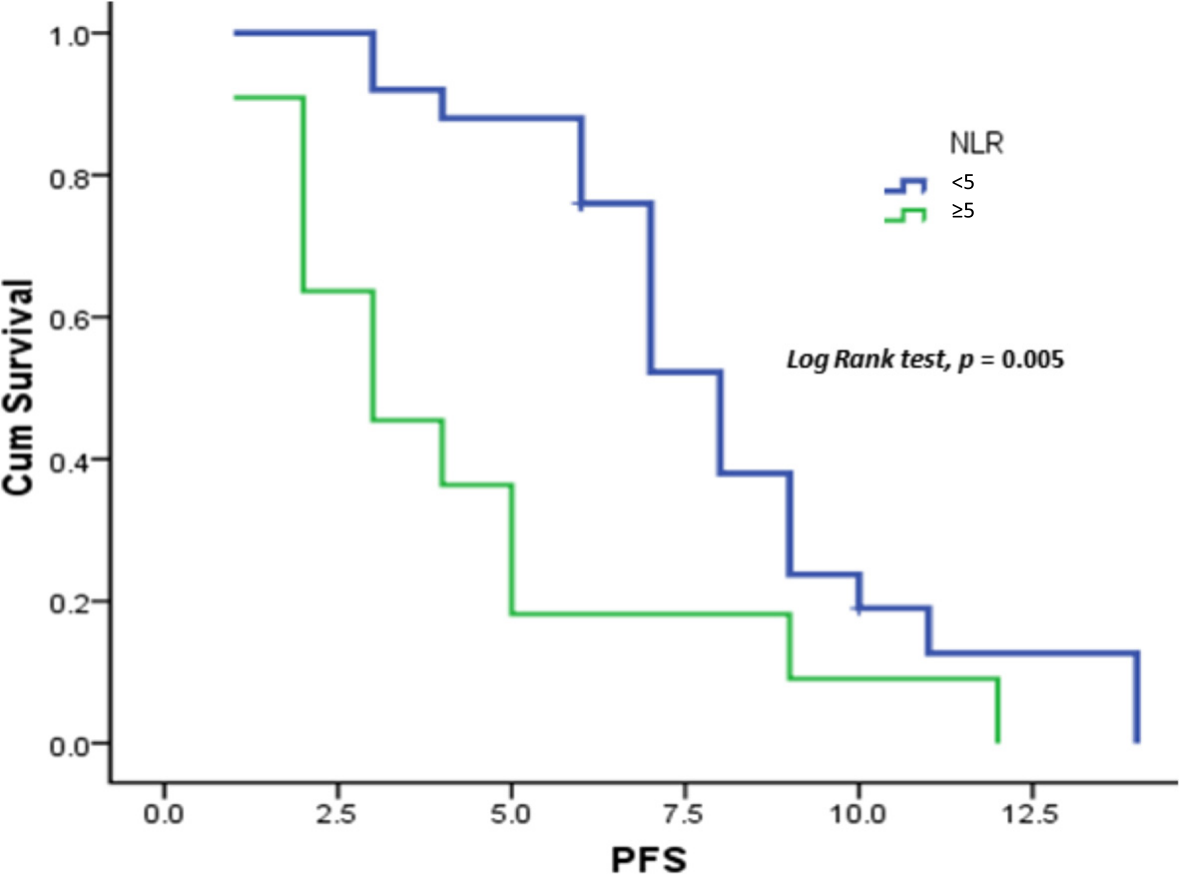
Progression free survival according to neutrophil to lymphocyte ratio (NLR)

**Fig. 3:**
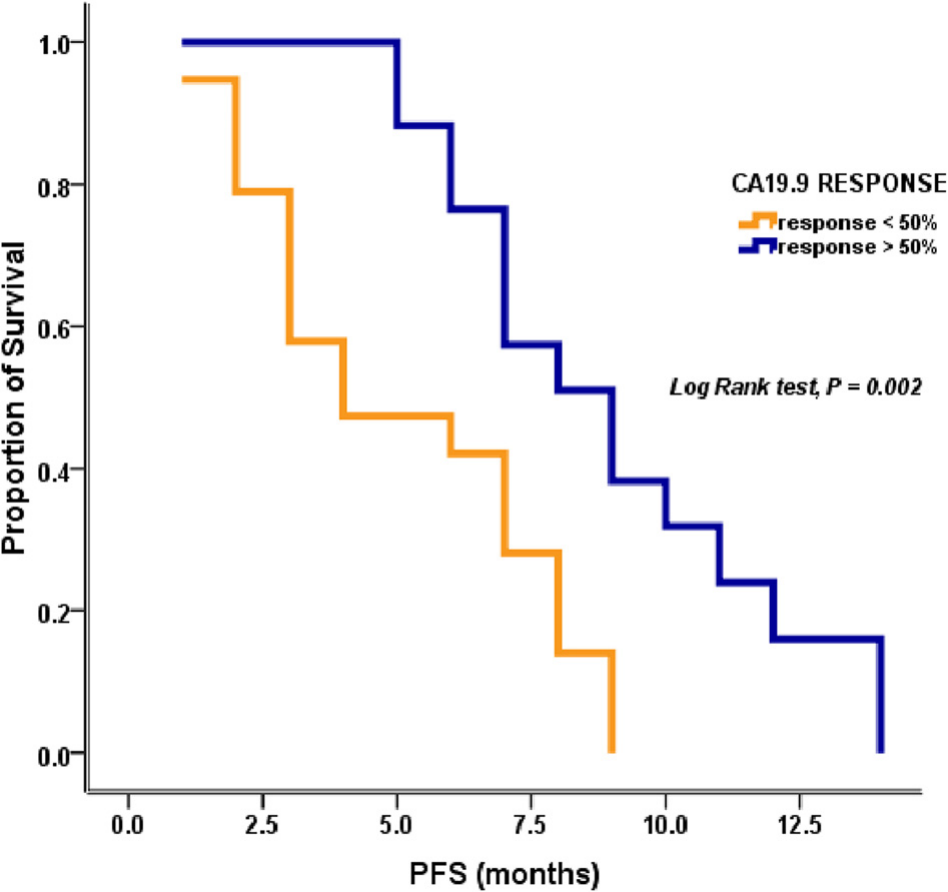
PFS according to Ca 19.9 response

At the time of data censoring, 27 patients had disease progression or had died; median PFS was 6.7 months (95 % CI 5.966–8.034) (Fig. 1). Multivariate analysis showed NLR ≥5 as an independent, negative, prognostic indicator (hazard ratio 3.845; 95 % CI 1.611–9.180; *p* = 0.002). Conversely, 12-week CA 19–9 decrease ≥50 % from baseline was demonstrated to be an independent, positive, prognostic factor (hazard ratio 0.274; 95 % CI 0.09–0.689; *p* = 0.006). Median PFS of patients with NLR <5 and NLR ≥5 was 8 and 3 months, respectively (*p* = 0.005) (Fig. 2). Median PFS was 4 months for patients with CA 19–9 decrease <50 % and 9 months for those with CA19-9 decrease ≥50 % (*p* = 0.007) (Fig. 3).

**Fig. 4:**
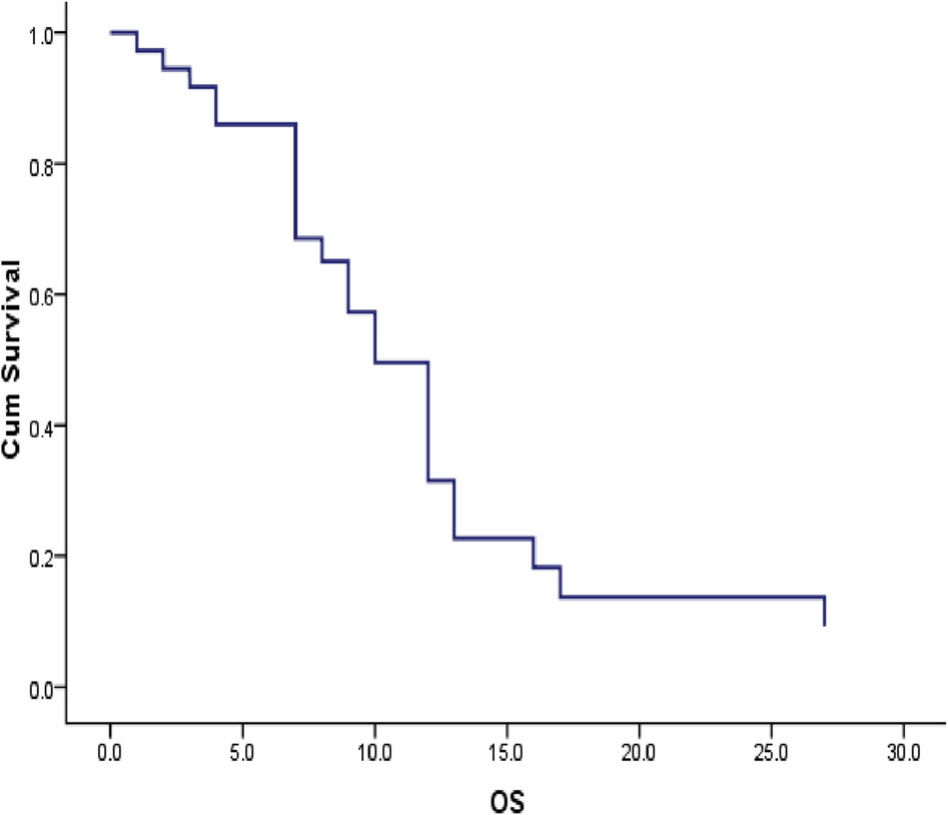
OS

**Fig. 5:**
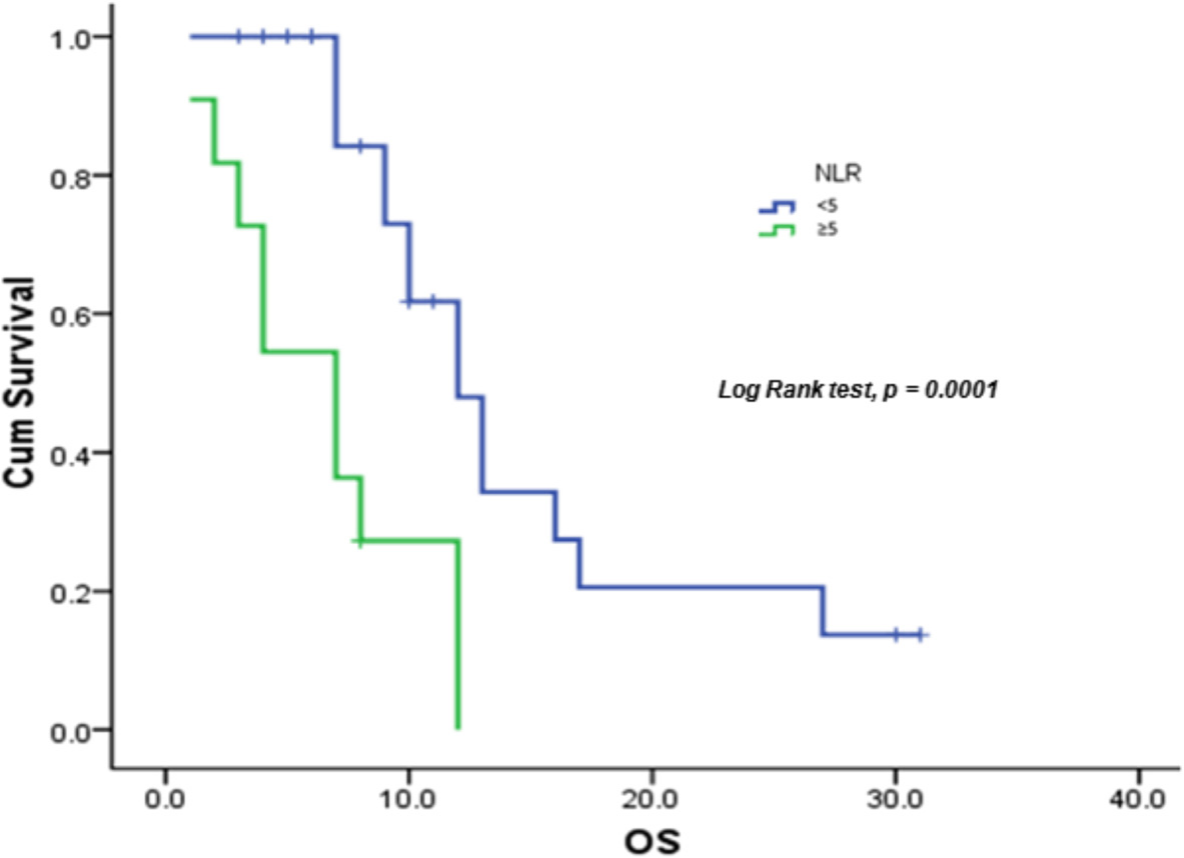
Overall survival according to neutrophil to lymphocyte ratio (NLR)

Survival analysis was based on 19 deaths (46 %). Median OS was 10 months (95 % CI 7.864–12.136), with a 12-month OS rate of 30.8 % (Fig. 4). Three patients (7.3 %) were still alive at 24 months after starting chemotherapy. OS was 12 and 7 months for patients with NLR <5 and ≥5, respectively (*p* = 0.0001) (Fig. 5). Furthermore, 12-week CA 19–9 decrease ≥50 % from baseline was significantly correlated with better PFS (*p* = 0.006). NLR ≥5 was the only variable showing a significant correlation with OS on univariate analysis (*p* = 0.002) (Tables 3 and 4).

**Table 3:** Multivariate analysis PFS

MULTIVARIATE ANALYSIS (PFS)			
	HR	95 % CI	*p*
KPS	2,18	0,8–5,9	0,125
CA19.9 RESPONSE	0,27	0,1–0,68	0,006
NLR ≥ 5	3,84	1,6–9,2	0,002
BILIRUBIN	1,4	0,59–3,3	0,444

**Table 4:** Univariate analysis OS

UNIVARIATE ANALYSIS (OS)			
	HR	95 % CI	*p*
AGE	0,78	0,32–1,89	0,6
SEX	0,63	0,28–1,41	0,3
TUMOR SITE	1,2	0,53–2,72	0,6
LIVER METASTASIS	1,61	0,67–3,8	0,3
MULTIPLE METASTASIS	0,9	0,35–2,3	0,8
KPS	1,9	0,58–6,2	0,3
BILIARY STENT	1,13	0,44–2,9	0,8
BILIRUBIN	1,8	0,8–4	0,1
CA19.9 BASELINE	1,75	0,62–4,9	0,3
CA19.9 DECREASE	0,59	0,25–1,37	0,2
NLR ≥ 5	4,3	1,7–10,8	0,002

### Safety

Treatment was well tolerated, and most toxicity was grade 1 or 2. No grade 4 events were recorded. Grade 3 toxicity comprised neutropenia in 10 patients (24.3 %), thrombocytopenia in five (12 %), anemia in three (7.3 %), diarrhea in four (9.7 %), nausea and vomiting in two (4.9 %), and fatigue in six (14.6 %). None of the patients discontinued treatment because of unacceptable toxicity; however, 20 % dose reduction of Nab-P alone, gemcitabine alone, or both drugs was needed in three (7.3 %), one (2.4 %), and two (4.9 %) patients, respectively (Table 5). Questionnaires on quality of life, before treatment and at each re-evaluation, were administered to all patients, yielding a 6-month CBR of 51.2 %, with 77.8 % of patients reporting an improvement in KPS.

**Table 5:** Toxicity

AE nab-P + Gem	
Grade 3 hematologic AE	no (%)
Anemia	3 (7.3 %)
Neutropenia	10 (24.3 %)
Trombocytopenia	5 (12.1 %)
Grade 3 non hematologic AE	no (%)
Fatigue	6 (14.6 %)
Peripheral Neuropathy	5 (12.2 %)
Diarrhea	4 (9.7 %)
Nausea	2 (4.9 %)

## Discussion

Prospective, randomized, phase III trials provide the best available evidence for comparison of different cancer treatments, to be eventually used as new standards of care in clinical practice. However, patients enrolled in clinical trials do not always fully mirror the characteristics of a real-life population. Thus, each novel oncological treatment needs to be assessed in daily clinical practice. According to the results of the randomized phase III MPACT trial, the addition of Nab-P to gemcitabine in patients with metastatic pancreatic cancer yielded significant clinical improvements in all endpoints across all subgroups, compared with gemcitabine alone [12]. Despite the limitations of a small series, we were able to confirm efficacy of Nab-P and gemcitabine in a cohort of patients observed in routine clinical practice. Specifically, we recorded a median OS of 10 months, median PFS of 6.7 months, and ORR of 26.7 %.

These data are even more significant when taking into account that a higher proportion of patients in our study was aged >70 years and had a KPS of 60–70 % compared with the population of the pivotal study (MPACT trial). Although pancreatic cancer mostly affects elderly people, clinical trials often include disproportionately fewer elderly patients than is commonly encountered in everyday clinical practice; furthermore, older age commonly leads to under-treatment in routine practice [18, 19]. This behavior is not justified by the results of retrospective studies, which showed no differences in outcome between older patients treated with palliative chemotherapy and younger patients enrolled in clinical trials [20]. In contrast, poor KPS is recognized as a major independent prognostic factor for OS in all patients with pancreatic cancer [21, 22]. In the MPACT trial, only 10 % of patients were older than 75 years and only 8 % had a KPS of 60–70 % [12]. Conversely, 26.8 % of our patients were older than 70 years, while 22 % had a KPS of 60–70 %. In our study, median PFS and median OS were higher than in the MPACT trial, although 22 % of patients had KPS of 60–70 % and the long-term analysis in the MPACT trial showed no survival benefit with gemcitabine and Nab-P in this subgroup of patients [23].

A potential bias seen in our retrospective study could be the timing of our assessment, namely, 12 weeks. However we used this timing according to our clinical practice. There is no standard second-line treatment following failure of first-line chemotherapy; however, second-line treatment does provide a survival benefit when compared with best supportive care [24–26]. Sixty-one percent of our patients received second-line treatment, mostly with capecitabine and oxaliplatin; therefore, we cannot rule out the possibility that the OS rate recorded in our study was affected by subsequent chemotherapy. However, the availability of an effective first-line regimen, Nab-P and gemcitabine, could allow maintenance of a better KPS, thus increasing the chance of exposing patients to subsequent treatment lines, with consequent benefit in terms of OS.

Of note, the safety profile in our study was concordant with that reported in the pivotal trial. The regimen was well tolerated and no patient required treatment discontinuation. Adverse events were generally grade 3 or lower and no grade 4 hematological or non hematological toxicity was observed. Although peripheral neurotoxicity was reported in 12.2 % of our patients, consistent with data reported in the MPACT study, it was rapidly reversible in most patients, with temporary discontinuation of Nab-P and subsequent dose reduction.

Among the prognostic factors related to PFS and OS evaluated in our study, CA19-9 levels and NLR >5 were of particular value. A CA19-9 decrease from baseline of >50 % turned out to be a positive independent prognostic factor related to PFS. Specifically, median PFS of patients with a decrease from baseline of <50 % was 3 months, while patients with a decrease of >50 % had a median PFS of 9 months. NLR >5 was shown to correlate negatively with PFS. Patients with NLR <5 had a median PFS of 8 months, whereas patients with NLR >5 had a median PFS of 3 months. NLR ≥5 was the only variable showing a significant correlation with OS on univariate analysis. Therefore, in accordance with the results of the MPACT trial, our study confirmed both serum levels of CA 19–9 and NLR as outcome predictors in patients with metastatic pancreatic cancer. In particular, NLR prognostic value points to the relevance of systemic inflammatory response in affecting outcome in patients with advanced pancreatic cancer [27, 28].

## Conclusions

After years in which the therapeutic options for metastatic pancreatic cancer have been scarce, FOLFIRINOX and Nab-P plus gemcitabine now represent two new options for treatment of metastatic pancreatic adenocarcinoma. In particular, our results confirm the activity, efficacy and tolerability of gemcitabine plus Nab-P as standard first-line treatment in patients with metastatic pancreatic cancer.

## Abbreviations

ALT: Alanine amino transferase
AST: Aspartate amino transferase
CBR: Clinical benefit rate
CI: Confidence interval
CR: Complete response
DCR: Disease control rate
G: Gemcitabine
HR: Hazard ratio
KPS: Karnofsky performance status
Nab-P: Nab-Paclitaxel
NLR: Neutrophil to lymphocyte ratio
ORR: Objective response rate
OS: Overall survival
PDAC: Pancreatic ductal adenocarcinoma
PFS: Progression free survival
PR: Partial responses
